# MtDNA diversity among four Portuguese autochthonous dog breeds: a fine-scale characterisation

**DOI:** 10.1186/1471-2156-6-37

**Published:** 2005-06-22

**Authors:** Barbara van Asch, Luísa Pereira, Filipe Pereira, Pedro Santa-Rita, Manuela Lima, António Amorim

**Affiliations:** 1 Instituto de Patologia e Imunologia Molecular da Universidade do Porto (IPATIMUP), Portugal; 2 CaniSemen Lda, Portugal; 3 Centro de Investigação em Recursos Naturais (CIRN), Universidade dos Açores, Portugal; 4 Faculdade de Ciências, Universidade do Porto, Portugal

## Abstract

**Background:**

The picture of dog mtDNA diversity, as obtained from geographically wide samplings but from a small number of individuals per region or breed, has revealed weak geographic correlation and high degree of haplotype sharing between very distant breeds. We aimed at a more detailed picture through extensive sampling (n = 143) of four Portuguese autochthonous breeds – Castro Laboreiro Dog, Serra da Estrela Mountain Dog, Portuguese Sheepdog and Azores Cattle Dog-and comparatively reanalysing published worldwide data.

**Results:**

Fifteen haplotypes belonging to four major haplogroups were found in these breeds, of which five are newly reported. The Castro Laboreiro Dog presented a 95% frequency of a new A haplotype, while all other breeds contained a diverse pool of existing lineages. The Serra da Estrela Mountain Dog, the most heterogeneous of the four Portuguese breeds, shared haplotypes with the other mainland breeds, while Azores Cattle Dog shared no haplotypes with the other Portuguese breeds.

A review of mtDNA haplotypes in dogs across the world revealed that: (a) breeds tend to display haplotypes belonging to different haplogroups; (b) haplogroup A is present in all breeds, and even uncommon haplogroups are highly dispersed among breeds and continental areas; (c) haplotype sharing between breeds of the same region is lower than between breeds of different regions and (d) genetic distances between breeds do not correlate with geography.

**Conclusion:**

MtDNA haplotype sharing occurred between Serra da Estrela Mountain dogs (with putative origin in the centre of Portugal) and two breeds in the north and south of the country-with the Castro Laboreiro Dog (which behaves, at the mtDNA level, as a sub-sample of the Serra da Estrela Mountain Dog) and the southern Portuguese Sheepdog. In contrast, the Azores Cattle Dog did not share any haplotypes with the other Portuguese breeds, but with dogs sampled in Northern Europe. This suggested that the Azores Cattle Dog descended maternally from Northern European dogs rather than Portuguese mainland dogs. A review of published mtDNA haplotypes identified thirteen non-Portuguese breeds with sufficient data for comparison. Comparisons between these thirteen breeds, and the four Portuguese breeds, demonstrated widespread haplotype sharing, with the greatest diversity among Asian dogs, in accordance with the central role of Asia in canine domestication.

## Background

Canine mtDNA sequence comparisons have been used to clarify the origins of the domestic dog [1,2], assess hybridisation with wild species [3,4], and forensic analysis [5,6]. Savolainen *et al *[1] and a recent revision aiming at nomenclature standardisation necessary for efficient data-basing [7], provided a good state of the art with a total of 1146 individuals studied, the majority assigned to a specific breed. However, the number of individuals per breed rarely exceeded 10. Most of the sequences reported surveyed the D-loop region between positions 15458 to 16039, a segment 582 bp long, of which 96 were polymorphic (72 transitions, 13 transversions and 14 indels), defining a total of 139 haplotypes. Six haplogroups were recognised (A to F), the first five interspersed with wolf lineages. Haplogroup A accounted for 70% of the lineages, and D to F just for 5%. As thorough as these studies were, there were still gaps and bias. In Europe, for instance, most studied breeds were British or Scandinavian, while mainland countries remained essentially unscreened.

This study intends to fill one of these gaps, i. e. the analysis of mtDNA lineages of breeds in the south western edge of Europe. At present, 10 Portuguese dog breeds are officially recognised by the Fédération Cynologique Internationale. This study focused on 4 of these indigenous breeds: the Castro Laboreiro Dog (Cão de Castro Laboreiro) in the Northwest, the Portuguese Sheepdog (Cão da Serra de Aires) in the South, the Serra da Estrela Mountain Dog (Cão da Serra da Estrela) in the Centre, and the Azores Cattle Dog (Cão de Fila de S. Miguel), in the Azores Islands. The Castro Laboreiro Dog is a mastiff type lupoid breed traditionally used in sheep herding and guarding, especially against wolves. It is thought to be one of the most ancient dog breeds of Iberia. The region of Castro Laboreiro is a secluded and remote area of the country that was not easily accessed until recent times. Therefore, it is possible that the breed has existed under isolation for a long period. The Serra da Estrela Mountain Dog is a molossoid breed thought to have been used for farm and herd guarding against wolves since ancestral times and its origin is also unknown. Two types of coat (long and short) have developed over time. The Portuguese Sheepdogs are thought to descend from a pair of Briard Sheepdogs, imported 100 years ago from France to Portugal. They are used in the Alentejo region for guarding and driving herds. The Azores Cattle Dog originated in São Miguel Island, where it is still used in cattle and property guarding. They are thought to descend from dogs brought into the island by Portuguese, Spanish, Flemish and French colonisers and travellers since the XV century. The breed is referred to since the beginning of the 20^th ^century. To different extents, all 4 breeds have experienced decreasing populations and have lost popularity over time. This is mostly due to the alteration of agricultural practices that rendered working dogs less useful, and to the introduction of competitive exotic breeds. These indigenous breeds are now winning back attention and their status is generally improving. Nevertheless, they still comprise relatively small population sizes, with registers at the Portuguese Kennel Club ranging between 96 Castro Laboreiro and 763 Serra da Estrela Mountain individuals in 2003. We tried to avoid one of the possible sampling biases by analysing a significant proportion of the animals registered in the studied breeds and we selected dogs from several kennels in order to minimise the relatedness of individuals and survey the internal diversity of the breed.

## Results & discussion

### MtDNA diversity in four Portuguese dog breeds

**Figure 1:**
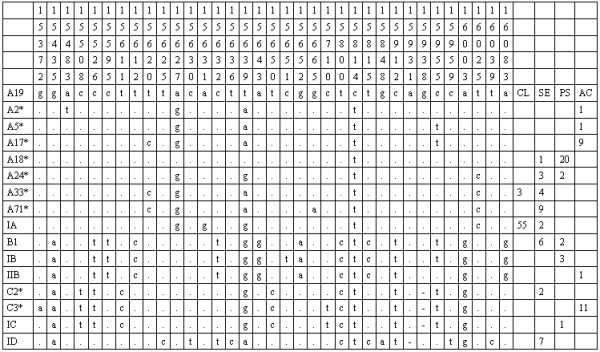
**Sequence alignments showing base substitutions and indels in the mtDNA D-loop region (582 bp) of 143 Portuguese dogs. **Sequence codes and numbers are given in the first column, with asterisks for previously published sequences. Only variable sites are shown, with sequence positions given above. Nucleotide positions are numbered according to Pereira *et al *(2004). Identity with the reference sequence (A19) is denoted by a stop, substitution by a different base letter, and deletions by a dash. Last four columns indicate the number of individuals in each breed (CL-Castro Laboreiro Dog; SE-Serra da Estrela Mountain Dog; PS-Portuguese Sheepdog; AC-Azores Cattle Dog).

**Figure 2:**
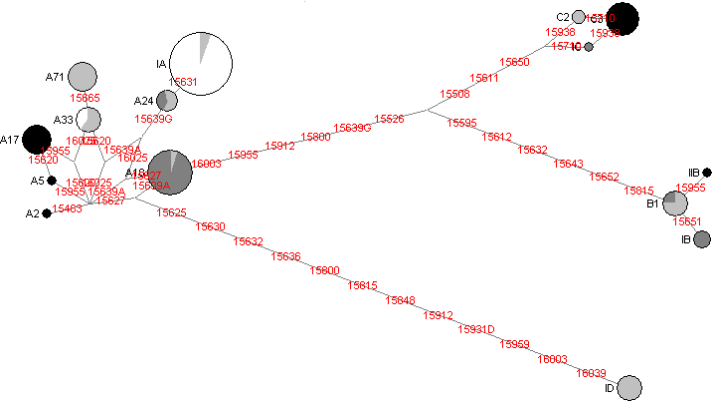
**Median-joining network of mtDNA haplotypes observed in four Portuguese dog breeds. **Circle sizes are proportional to the number of individuals they represent. White, light grey, dark grey, and black represent Castro Laboreiro Dog, Serra da Estrela Mountain Dog, Portuguese Sheepdog and Azores Cattle Dog, respectively.

A total of 15 different haplotypes was found in 143 dogs, five of them newly described (Fig. 1). These haplotypes belong to the four major haplogroups A, B, C and D and their frequencies were 77%, 8%, 10% and 5%, respectively. The considerably higher frequency of haplogroup A in Portugal, when compared to the 68% observed in Europe by Savolainen *et al *[1], results from the 100% prevalence observed in the Castro Laboreiro Dog, as discussed below. The median-joining network (Fig. 2) displaying the Portuguese haplotypes shows high diversity between haplotypes belonging to different haplogroups, and also between haplotypes inside the same haplogroup, particularly in haplogroup A, thus indicating inputs from different founders rather than local population expansions.

Among the four breeds studied here (Table 1), the Castro Laboreiro Dog (n = 58) presented the most differentiated history. This breed has the lowest level of diversity, as only two A haplotypes were found. Strikingly, a new A haplotype (IA), one step apart from haplotype A24 (a 15631A → G transition), is shared by 95% of the sample. Haplotype IA was also observed in two Serra da Estrela Mountain dogs. The remaining 5% display haplotype A33, also present in four Serra da Estrela Mountain individuals. Haplotype A33 was previously described only in one Sloughi dog from North Africa [1], two Retrievers and one unspecified dog [10].

**Table 1:** Summary statistics of diversity measures for 13 dog breedsDiversity measures for 4 Portuguese breeds and 13 other for which at least 10 individuals have been typed in previous studies [1,10,11,12].

Breed	n	Number of		Haplotype diversity	Mean n° of pairwise differences	Nucleotide diversity
		haplotypes	haplogroups			

Castro Laboreiro Dog	58	2	1	0.100 ± 0.052	0.299 ± 0.317	0.001 ± 0.001
Azores Cattle Dog	23	5	3	0.640 ± 0.065	7.012 ± 3.418	0.012 ± 0.007
Portuguese Sheepdog	28	5	3	0.484 ± 0.110	4.780 ± 2.408	0.008 ± 0.005
Serra da Estrela Mountain Dog	34	8	4	0.852 ± 0.030	9.802 ± 4.601	0.017 ± 0.009
German Shepherd ^[1,12]^	16	3	2	0.575 ± 0.112	4.500 ± 2.338	0.008 ± 0.004
Retriever Labrador ^[1,10]^	22	7	2	0.771 ± 0.060	4.554 ± 2.327	0.008 ± 0.004
Basenji ^[1]^	10	3	1	0.644 ± 0.101	2.156 ± 1.304	0.004 ± 0.003
Akbasch^[1]^	11	5	2	0.618 ± 0.164	3.455 ± 1.909	0.006 ± 0.004
Canaan Dog^[1]^	17	6	2	0.831 ± 0.056	7.801 ± 3.821	0.013 ± 0.007
Saluki ^[1,11]^	16	8	2	0.808 ± 0.093	7.875 ± 3.867	0.014 ± 0.007
Kangal^[1]^	10	5	4	0.822 ± 0.097	9.622 ± 4.823	0.017 ± 0.009
Ryukyu ^[11,12]^	14	7	3	0.879 ± 0.058	8.924 ± 4.377	0.015 ± 0.008
Shiba ^[11]^	28	8	3	0.791 ± 0.049	6.717 ± 3.265	0.012 ± 0.006

**Figure 3:**
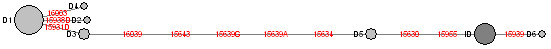
**Median-joining network of dog mtDNA haplotypes belonging to haplogroup D. **Haplotypes D1, D2, D3 and D4 were observed in Scandinavian dogs, while D5 was observed in Turkey, D6 in Spain and ID in this work. Circle sizes are proportional to the number of individuals they represent.

All major haplogroups were observed in Serra da Estrela Mountain dogs (n = 34). The breed presents the highest values of diversity measures, implying that it harbours a comparatively more variable gene pool. It shares five out of its eight haplotypes with other breeds, namely two with the Castro Laboreiro Dog (A33 and IA) and three with the Portuguese Sheepdog (A18, A24 and B1). The breed also displays a new haplotype (ID) belonging to haplogroup D, this latter being common in Scandinavia but rarely observed elsewhere. D haplotypes were previously described in eight Scandinavian breeds (n = 18), two Kangal Dogs (Turkey) and one Galgo Español (Spain) [1]. Newly reported ID was also found in seven Serra da Estrela Mountain dogs. However, it is closer to Turkish D5 and Spanish D6 (respectively 2 and 1 mutational steps apart) than to Scandinavian D1, D2, D3 and D5 (Fig. 3). Clearly, genetic inputs from diverged populations must have repeatedly enriched the genetic diversity of the Serra da Estrela Mountain dogs.

The Portuguese Sheepdog (n = 28) presented intermediate levels of diversity when compared to the Castro Laboreiro Dog and the Serra da Estrela Mountain Dog, mainly because one haplotype (A18) was present in 71% of the sample. This haplotype has a worldwide distribution and was also found in one Serra da Estrela Mountain dog. Haplotype A24 is also shared with the Serra da Estrela Mountain breed and was previously reported in British (n = 4) and Pyreneean breeds (n = 3). Haplotype B1, also present in the Serra da Estrela Mountain Dog, was observed in two individuals. A new B haplotype (IB), one mutational step apart from haplotype B1 (a 15651C → T transition), so far exclusive to the breed, was also observed in three dogs. A new C haplotype (IC), one mutational step apart from haplotype C3 (a 15672G → A transition), was displayed by one individual.

The Azores Cattle Dog (n = 23), an insular Portuguese breed, presented the second highest levels for diversity measures following the Serra da Estrela Mountain Dog, but did not share lineages with the other breeds surveyed in mainland Portugal. A single individual presented a newly observed haplotype (IIB), one-step apart from haplotype B1 (a 15955T → C transition), which is widely found in Europe but was not observed in this breed. Nearly half of the individuals (n = 11) presented haplotypes that are more frequent in the northern region of Europe (A2, A5 and A17), and quite differentiated from the other A haplotypes found in the other Portuguese breeds (Fig. 2). The remaining individuals display haplotype C3, which was also reported in North Europe and in Asia [1,6,11].

### Comparative analysis of mtDNA haplotype structure

We collected published data on all breeds for which at least ten individuals were typed [1,10-12]. Only 13 breeds met this criterion: two European (German Shepherd and Retriever Labrador), four Middle-Eastern (Akbasch, Canaan Dog, Saluki and Kangal), two Asian (Ryukyu and Shiba), one African (Basenji) and the four breeds here described. Although these numbers are still undesirably low, they can nevertheless provide insights into the apportionment of overall domestic dog mtDNA diversity in terms of intra-and interbreed components. The summary statistics of diversity measures are shown in Table 1. Regarding haplotype diversity, we observed that all breeds but the Castro Laboreiro Dog and marginally the Portuguese Sheepdog, showed values above 0.5, which immediately reveals considerable intra-breed heterogeneity. This heterogeneity, however, could be due to slight nucleotide differences: it could be that different haplotypes within a breed were assigned to the same haplogroups, which in turn were not shared across breeds. This is not the case, for (a) only two breeds (Castro Laboreiro Dog and South African Basenji, although in the latter only 10 individuals were typed) show haplotypes belonging to the same haplogroup and (b) haplogroup A is present in all breeds and haplogroup B, the second most frequent haplogroup, is shared by several breeds from different continental areas. Even the relatively infrequent haplogroup C shows substantial sharing among breeds. In relation to haplogroups D and E, due to their overall rarity, it is premature to speculate.

**Figure 4:**
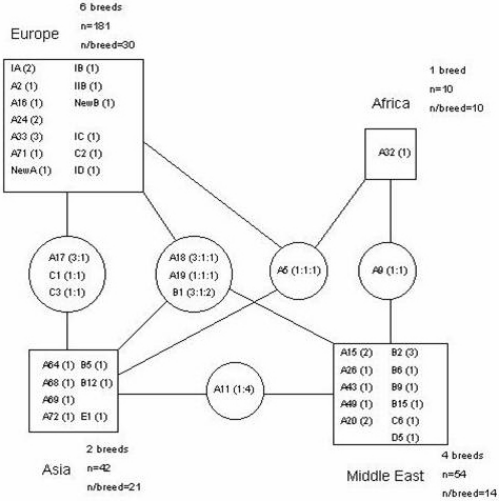
**MtDNA haplotype sharing between dog breeds and world regions. **Thirteen breeds for which at least 10 individuals were typed are included. Squares represent world regions and state haplotypes observed therein. Figures inside brackets indicate the number of breeds where the particular haplotype was observed. Circles contain haplotypes shared between breeds from different regions; numbers enclosed in parenthesis are to be read from right top to left down, and correspond to the number of breeds in which a haplotype is observed in each continental region. For example, "A18 (3:1:1)" indicates that haplotype A18 was found in 3 European, 1 Asian and 1 Middle-Eastern breeds.

Still more remarkable is the fact that the number of haplotypes shared between breeds inside a region is generally lower than the number of haplotypes shared between breeds from different regions (Fig. 4). In Europe the figures are, respectively, 6 and 7; in the Middle East, 5 and 5; and in Asia, 0 and 8 shared haplotypes. These values are still debatable due to the low number of individuals typed per breed and the limited number of breeds analysed so far, particularly in Africa and America. Nonetheless, it indicates that many of the extant domestic dog breeds, despite their phenotypic homogeneity, are substantially heterogeneous in their maternal ancestry.

In order to quantify the partition of the overall diversity into separate components, we performed AMOVA in two ways: (a) when considering all the 13 breeds as a single group, 32.80% of the variation was found to be due to inter-breed diversity and 67.30% was attributable to the intra-breed component; (b) when considering the breeds grouped in 4 worldwide regions, the resulting values were remarkably similar: 29.51% for inter-breed; 66.02% for intra-breed; and 4.47% for diversity between regions. These values demonstrate that the geographic component of the domestic dog mtDNA diversity, at least at the broad continental level, is very low.

Almost all pairwise genetic distances (measured as F_ST_s) between the 13 breeds were statistically significant (data not shown) except for the pairs Azores Cattle Dog (Europe)-Kangal (Middle East) (*p *= 0.063); Portuguese Sheepdog (Europe)-Ryukyu (Asia) (*p *= 0.063); Saluki (Middle East)-Canaan Dog (Middle East) (*p *= 0.396); Canaan Dog (Middle East)-Ryukyu (Asia) (*p *= 0.180); Kangal (Middle East)-Ryukyu (Asia) (*p *= 0.117), again evidencing no geographical correlation.

## Conclusion

This fine-scale survey undertaken on four autochthonous Portuguese dog breeds revealed different histories, even for geographically close breeds with similar selection characteristics. The unprecedented low mtDNA diversity observed in Castro Laboreiro dogs contrasts with the high diversity displayed by Serra da Estrela Mountain dogs, both of them herding breeds with putative centres of origin just 200 km apart. The Castro Laboreiro Dog has clearly suffered a population bottleneck (although the hypothesis of a low number of female lineages at its origin can not be excluded) that severely reduced its mtDNA diversity. In contrast, the Serra da Estrela Mountain Dog mtDNA diversity shows that the breed has not remained in isolation. This may also indicate that if population bottlenecks have occurred in this breed, subsequent expansions included dogs from other origins.

A peculiar pattern of haplotype sharing between the Portuguese breeds was also detected: just between the Serra da Estrela Mountain Dog (with putative origin in the centre of the country), and the breeds to its north and south. The Serra da Estrela Mountain Dog presented two haplotypes in common with the north-western Castro Laboreiro breed (which behaves, at the mtDNA level, as a sub-sample of the Serra da Estrela Mountain breed) and three with the southern Portuguese Sheepdog breed.

It would be interesting to compare the Portuguese Sheepdog mtDNA haplotypes with those of the Briard Sheepdogs, from which they are supposedly descending. Contrastingly, the insular Azores Cattle Dog did not share any haplotypes with continental Portuguese dogs, but mostly with dogs from Northern Europe. This suggests that the Azores Cattle Dog could descend maternally from Northern European rather than Portuguese mainland dogs, which is consistent with the historical reports.

When comparing breeds from different regions at a worldwide scale, it is Asia that harbours most of the haplotype sharing, in accordance with the postulated domestication centre [1]. The broad geographic pattern of haplotype sharing is, however, paradoxical, because haplotype sharing is just as likely to occur between breeds from the same continent as between breeds from distant ones.

The observation of molecularly distant haplotypes within most breeds indicates recent and polyphyletic origins for the majority of female gene pools of the current breeds.

Although it is clear that mtDNA haplotypes have poor breed discriminating power, they are valuable for determining the origin and population history of dog breeds. We expect that the data we present here and further developments will allow for better resolution of the population histories of European dog breeds, since they are still poorly sampled.

## Methods

### Sample collection and DNA extraction

A total of 143 individuals were selected for the study based on breed assignment (Azores Cattle Dog, n = 23; Castro Laboreiro Dog, n = 58; Portuguese Sheepdog, n = 28; Serra da Estrela Mountain Dog, n = 34) excluding first order relatives in order to reduce sampling bias. A large number of individuals per breed (mean = 36) collected throughout the geographical areas of origin, also contributed to maximise the informative power of the sample.

Samples consisted in whole blood on FTA (Whatman) cards or buccal epithelium cell swabs collected with cytology brushes. DNA from blood samples was extracted according to standard Chelex 100 or phenol-chloroform methods. Buccal swab samples were treated with a standard salting-out extraction protocol.

### DNA amplification and sequencing

Two overlapping fragments of the mtDNA D-loop region were amplified so that a 582-bp fragment between positions 15458–16039 was surveyed. Polymerase chain reaction (PCR) was performed in 35 cycles after an initial denaturing step of 95°C for 2 min. For primer pair A (5'-TTA CCT TGG TCT TGT AAA CC-3' and 5'-CTG AAG TAA GAA CCA GAT GCC-3'), conditions were 95°C for 30 s, 58°C for 30s, and 72°C for 30s. For primer pair B (5'-CAT ACT AAC GTG GGG GTT AC-3' and 5'-CCA TTG ACT GAA TAG CAC CTT G-3'), conditions were 95°C for 30 s, 60°C for 30 s, and 72°C for 30s. A final extension step of 72°C for 10 min was performed in both cases. PCR mixture consisted of 2 μL of DNA extract, 0.25 μM of each primer, 1.5 mM of MgCl_2_, 0.2 mM of dNTPs, 67 mM Tris-HCl pH 8.8, 16 mM (NH_4_)_2_SO_4_, 0.01% Tween 20 and 0.5 units of Taq DNA Polymerase (Bioron) in a total volume of 12.5 μL. Negative controls were included (one in each PCR) to monitor for contamination and resulted in no amplification product. The reaction was run on a GeneAmp PCR System 2400 (Perkin Elmer). The sequencing reaction was performed on forward and reverse directions using 2.5 μL of purified PCR product, 0.25 μM of primer (A and B) and 2 μL of big dye terminator (Abi Prism Big Dye Terminator Cycle Sequencing Kit v3.1) for a total reaction volume of 5 μL. The cycle program consisted of an initial step of denaturation (96°C, 2 min), 35 cycles (96°C, 15 s; 50°C, 9 s, 60°C, 2 min) and a final extension step (60°C, 10 min). The reaction was run on a GeneAmp^® ^PCR System 2700 (Applied Biosystems). The cycle sequencing products were analysed on an ABI 3100 Automated Sequencer (Applied Biosystems) according to the manufacturer's directions.

### Data analysis

The sequences obtained were compared by alignment (Fig. 1) to the reference sequence [8], indicated as haplotype A19 (GenBank accession entry: NC_002008) and polymorphisms were numbered accordingly to Pereira *et al *[7]. Medium-spanning networks including previously published data were calculated using Network 4.0 , equally weighting polymorphisms. Diversity measures, AMOVA and F_ST _distances were obtained using Arlequin 2.0 software [9].

## Authors' contributions

BA carried out most of the sample processing, participated in the sequence alignment and drafted the manuscript. LP participated in the conception and co-ordination of the study, performed the statistical analysis and helped with subsequent drafts. FP participated in the sample processing and the sequence alignment. PS-R and ML sampled the dogs studied and provided background information. AA conceived the study, participated in its design and co-ordination and helped to prepare the final manuscript. All authors read and approved the final manuscript.
